# Tracking Changing Environments: Innovators Are Fast, but Not Flexible Learners

**DOI:** 10.1371/journal.pone.0084907

**Published:** 2013-12-31

**Authors:** Andrea S. Griffin, David Guez, Françoise Lermite, Madeleine Patience

**Affiliations:** School of Psychology, University of Newcastle, Callaghan, New South Wales, Australia; The Australian National University, Australia

## Abstract

Behavioural innovations are increasingly thought to provide a rich source of phenotypic plasticity and evolutionary change. Innovation propensity shows substantial variation across avian taxa and provides an adaptive mechanism by which behaviour is flexibly adjusted to changing environmental conditions. Here, we tested for the first time the prediction that inter-individual variation in innovation propensity is equally a measure of behavioural flexibility. We used Indian mynas, *Sturnus tristis*, a highly successful worldwide invader. Results revealed that mynas that solved an extractive foraging task more quickly learnt to discriminate between a cue that predicted food, and one that did not more quickly. However, fast innovators were slower to change their behaviour when the significance of the food cues changed. This unexpected finding appears at odds with the well-established view that avian taxa with larger brains relative to their body size, and therefore greater neural processing power, are both faster, *and* more flexible learners. We speculate that the existence of this relationship across taxa can be reconciled with its absence within species by assuming that fast, innovative learners and non innovative, slow, flexible learners constitute two separate individual strategies, which are both underpinned by enhanced neural processing power. This idea is consistent with the recent proposal that individuals may differ consistently in ‘cognitive style’, differentially trading off speed against accuracy in cognitive tasks.

## Introduction

Behavioural innovations -solutions to novel problems, or novel solutions to old problems [1]- are increasingly thought to provide a rich source of phenotypic plasticity and evolutionary change [2]–[4]. Innovation propensity shows substantial variation across avian taxa and the functional significance of such variation is well documented. The number of anecdotal reports of novel feeding behaviours in the wild, aka innovation rate [4], is correlated across avian taxa with a variety of ecological variables, including urbanization [5] but see [6], habitat degradation [7], introduction to novel environments [8]–[11], and seasonal resource variability in habitats of resident species [12]. This body of work indicates that the ability to innovate provides an adaptive mechanism by which avian species flexibly adjust to changing environmental conditions [4].

Innovation propensity varies not only across species, but also across individuals. In several species studied to date, individual differences in innovation tendency are stable across time [13]–[15], and have been found to be associated with different life history strategies and differential reproductive success [16]–[18]. Given the strong evidence that the prevalence of innovative behaviour at the taxon level is indicative of behavioural flexibility, it is reasonable to assume that variation at the individual level in innovation propensity should reflect inter-individual differences in flexibility. In other words, individuals with higher innovation propensity should be behaviourally more flexible and therefore able to adjust to changing environmental conditions more rapidly than individuals with lower innovation propensity. Yet, to our knowledge, this key prediction has not been tested to date.

Serial discrimination reversal learning is a well-established classic test of behavioural flexibility [19]–[22]. One particular version of this instrumental conditioning task requires responding to a food-rewarded cue (S+), and withholding from responding to a non-rewarded cue (S−). Once the S+/S− discrimination is learnt, the reward contingencies are reversed. This procedure is then repeated several times and the speed at which individuals learn the successive reversals yields a measure of how amenable individuals are to changing their behaviour as the environment changes. The validity of this measure to quantify cross species differences in flexibility has received some criticism because species differences may be attributable to extraneous variables, such as ability to adjust to captive conditions, that vary across species, but have little to do with reversal learning per se [23]–[25]. It has been suggested that within species comparisons may be less vulnerable to such confounding variables, however [26]. Reversal learning constitutes hence an independent measure with which to test the prediction that inter-individual variation in innovation propensity is a measure of behavioural flexibility.

Here, we tested the prediction that inter-individual variation in innovation propensity is a measure of behavioural flexibility using Indian mynas, *Sturnus tristis*, (formerly classified as *Acridotheres tristis* [27], and also referred to as the common myna), a highly successful worldwide invader. Mynas are highly adaptable and their high behavioural flexibility is well supported by a growing body of published scientific studies [28]–[34]. Mynas are hence an ideal species in which to explore the relationships between individual variation in innovation propensity and behavioural flexibility.

We used a novel extractive foraging task to measure innovative performance and a serial discrimination reversal learning task to measure behavioural flexibility. First, based on prior evidence that innovation propensity is positively correlated with learning speed in birds [35]–[37], we predicted that mynas that were faster to solve the extractive foraging task would learn the S+/S− discrimination faster. Second, in line with our prediction that innovation propensity and behavioural flexibility should be positively correlated, we predicted that mynas that solved the extractive foraging task faster would learn the cue reversals faster.

## Materials and Methods

### Ethics Statement

All animal care, husbandry, and experimental procedures were in accordance with the Australian code of practice for the care and use of animals for scientific purposes, and were approved by the University of Newcastle Animal Ethics Committee (protocol A-2011–154). No additional license is required to trap mynas, as they are classified as an introduced, invasive pest species.

### Subjects

Subjects were 18 wild-caught adult Indian mynas (7 females, 11 males). Birds were captured in Newcastle (NSW, Australia). Due to technical problems and one bird becoming unwell during the experiments, all 18 birds completed reversals 1–2, 16 mynas completed additional reversal 3, and 15 mynas completed all 4 reversals (see below).

Birds were captured using a walk-in baited trap specifically designed to trap this species [38]. This trap, which is described in detail elsewhere [30], works by allowing mynas to enter a bottom cage (1×1×1 m), collect a bait, fly up through two small (0.1 m diameter), one-way channels into a top cage (1×1×1 m), and rest on perches while consuming the food item. Given the natural tendency of this species to aggregate, surrounding mynas approach and enter the trap, attracted in particular by the contact calls of trapped birds. As a consequence, mynas accumulate in the top cage. The trap is equipped with an opaque roof and shaded sides, which provide birds with sun protection and cover. Small dog pellets, a preferred food of Indian mynahs, were provided ad libitum in both top and bottom cage, together with ad libitum water (for more details, see [30]). The trap was checked and emptied each day, and birds were transported in small cotton individual holding bags to the University of Newcastle Central Animal House in an air-conditioned vehicle.

Upon arrival they were weighed, measured and banded with individually identifiable plastic bands, and released into a large outdoor group aviary (length 4.4 m×width 1.25 m, and 2.25 m high). The aviary was equipped with perches, shelters and a large water bath. Mynas were left undisturbed for seven days to acclimatize to captivity. Birds had access to water and dog pellets ad libitum, except during innovation tasks, which required short periods of food deprivation. During innovation tests, birds also received dog pellets.

At the end of testing, birds were returned to the large outdoor group holding aviaries to take part in other ongoing studies in our laboratory.

### General Procedure

Over the 6-month period that followed bird capture, we obtained several measures of innovation performance for each bird using a variety of different extractive foraging tasks. The first two innovation measures were taken on two consecutive days, while the third measure was obtained between 6 weeks and 10 weeks later. Birds also completed a serial discrimination reversal learning test. The order in which the serial discrimination reversal learning test and the innovation tests was completed was counterbalanced across subjects.

### Innovation

**Figure 1:**
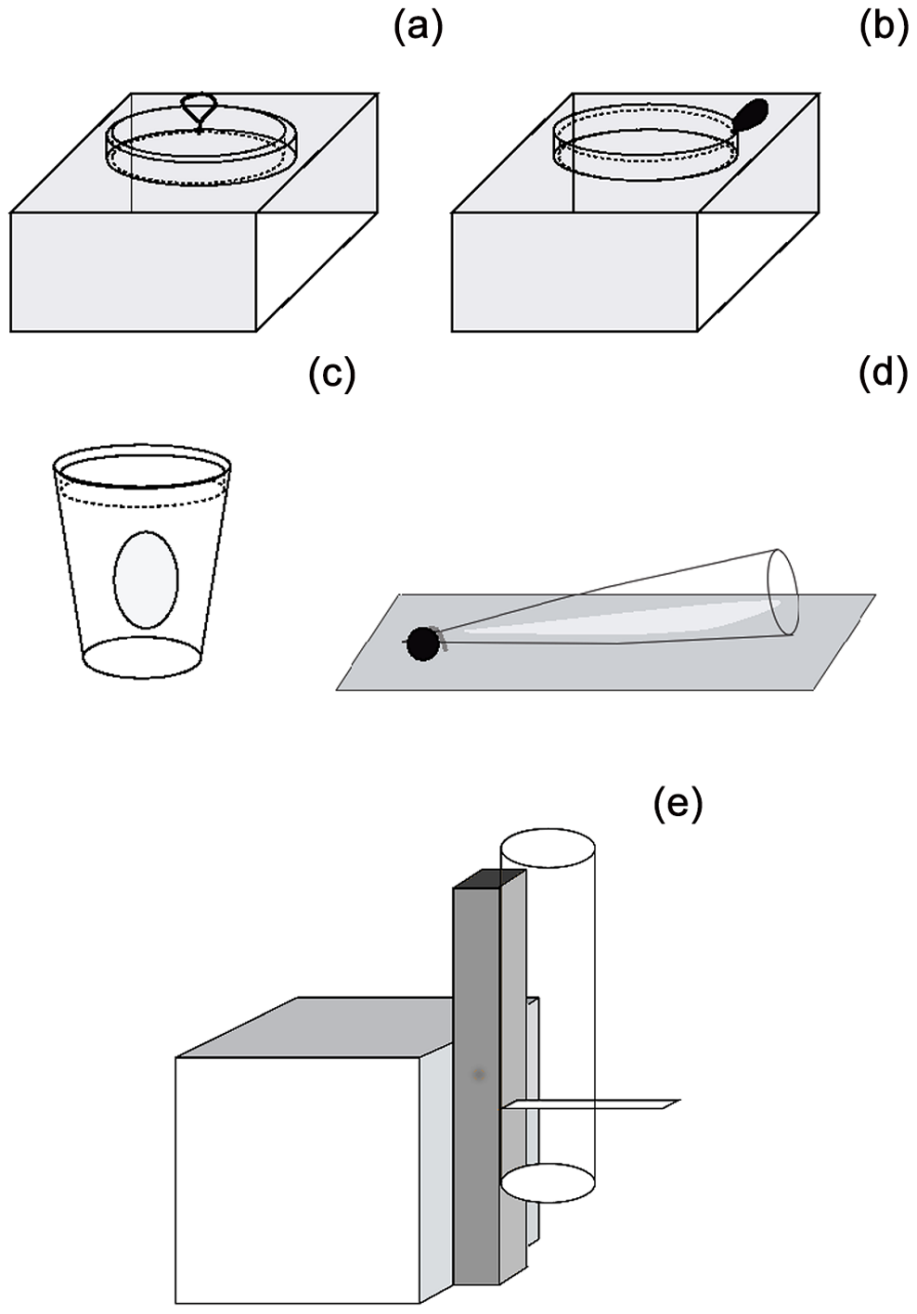
Schematic of innovation tasks. Each bird was tested on a pseudorandom selection of two tasks amongst those depicted in a–d. All birds were also tested on the task depicted in e. See text for more details.

Each bird was presented with two of four possible different novel extractive foraging tasks on the first two innovation trials, and a fifth task on the third innovation trial (Figure 1). Although this meant that different individuals received different tasks, our aim in analyzing the innovation performance in this way was to ensure that any relationship found between innovation performance and behavioural flexibility was independent of the particular innovation task used. Analyses revealed that there were no significant differences in performance across any of the tasks (see results). The first two possible tasks consisted of a Petri dish with either an inverted (Figure 1a), or an upright, lid (Figure 1b). The inverted lid could only be lifted by grabbing a hook attached to its center, while the upright lid could be removed by either leveraging it upwards, or grabbing a piece of tape attached to its edge. The third possible task consisted of a Styrofoam coffee cup glued to a small wooden board (Figure 1c). The cup was covered with a Petri dish lid, which was glued into place so it could not be removed, but allowed visual access to the food inside the cup. One 3 cm diameter hole in the side of the cup was covered in transparent plastic film, which needed to be pierced to access the food. The fourth task consisted of a piece of paper that needed to be pulled out of a plastic champagne flute to access the food (Figure 1d). The fifth task was a 3 cm diameter, 14 cm long transparent vertical tube attached to a stand (Figure 1e). A thin (0.5 mm) plastic flap (3×5 cm) was inserted horizontally half way up the tube, so that food inside the tube was trapped, but fell down the tube on to the ground when the flap was pulled. Neophobia responses to the tasks were reduced by presenting the task to the birds on the evening before the test with a few dog pellets either in the open container (Petri dish tasks) or beside the container (cup, flute and tube). In this way, all birds had 3–4 h exposure to each task in the evening and 1–2 h exposure to it in the morning prior to the innovation test. All birds had consumed the readily available food from the task prior to the start of each innovation test.

For testing, each bird was transferred to an individual test aviary (length 2 m×width 1 m, and 2 m high) and allowed two days to acclimatize. Birds were food-deprived 1–2 h before sunset (other than the few dog pellets available on the open innovation task left in the cage to reduce neophobia, see above), and tested the next morning within 1–3 h of sunrise. During all tests, the focal myna was filmed from behind an observation hide placed 6 m away from the aviary. To initiate an innovation test, the experimenter approached the focal bird from behind the hide, and placed a dog pellet beside the task before returning to the hide. This baseline trial ensured that the bird was motivated to feed. Once the focal subject had consumed the dog pellet, the experimenter approached once again from behind the hide, and placed a dog pellet inside the task before returning to the hide. The latency from first contact to solving the task was measured. Each trial lasted 30 min. Tests for which no solving occurred were attributed a capped latency of 1801 s. At the end of testing the birds were moved back to the flight aviary.

### Serial Discrimination Reversal Learning

#### Apparatus

For the serial discrimination reversal learning task, mynas were transferred to (length 60 cm×width 30 cm, and 60 cm high) home cages and housed there for the duration of the experiment. Individually-held birds were visually, but not acoustically isolated from each other, in order to facilitate adaptation to individual housing. Each home cage was equipped with several perches, a water tube, a pecking key and a food hopper. The pecking key could be backlit with either a white, blue, or red light. The food hopper contained dog pellets, which were accessible to the bird when the hopper was engaged and unavailable when it was disengaged. A request perch was fitted with an infra-red beam and was located approximately 15 cm in front of the food hopper. Another infra-red beam spanned the entrance of the food hopper.

All equipment and stimulus presentations were controlled automatically by a Med Associates PC-IV software program running on a computer in a room adjacent to the bird holding room. Performance was monitored continuously by the computer-controlled software, and each bird’s progression through different phases of the serial discrimination reversal learning task (preliminary training, discrimination training, reversal learning, see below) and different trial types (S+/S− reversals, see below) occurred automatically. This allowed us to test birds, and measure learning performance, continuously.

#### Preliminary training

Following transfer to the operant conditioning cages, birds were left for two days with the food hopper engaged so that they could become familiarized with the location of food in their new surroundings. Each bird then underwent preliminary training in which it was gradually shaped to 1. use the request perch to cause the pecking key to light up, and 2. peck the backlit key to engage the food hopper and hence gain access to food. When activated by a perch request, the pecking key switched on and remained lit for 10 s unless it was pecked. Pecking the key caused the key to switch off, and was rewarded by a 5-s access to the food hopper. The amount of food reward (dog pellets) each bird received on each trial was hence capped by access time to the feeder, and not fixed to a set quantity. During preliminary training, the pecking key was backlit with a white light.

Once an individual bird reliably used the perch to request a pecking key presentation, and pecked the key as soon as it lit up to gain access to the food hopper, it completed 80 trials (i.e. 80 pecking key presentations followed by key pecking and feeding from the hopper), after which the computer controlling the instrumental conditioning equipment automatically and immediately placed the bird on the discrimination learning task.

There was no other food available in the home cage, other than that provided by the key-triggered food hopper. Hence, birds obtained their entire daily food ration through operating the conditioning device. In this way, we ensured that birds completed learning trials based on their own motivation, without imposing any food deprivation. This motivation was expressed by the each bird’s own decision to land on the perch, hence self-requesting a pecking key presentation to gain access to the food hopper. Following a typical small weight loss immediately after being moved to individual housing and during preliminary training, birds’ weights typically re-increased to around their original weights measured at the time when they were first moved into the operant conditioning cages (±5%), and stabilized thereafter.

#### Discrimination acquisition

The initial discrimination consisted of a red-blue colour discrimination task. These two colours were selected on the basis of that they are highly discriminable for avian species [39]. The specific colour that served as the first S+ was counterbalanced across birds, and the order in which the S+ and S− were presented was random with the restriction that no more than two successive presentations of either cue occurred. Upon activation of the request perch, the S+ was presented for 10 s. Pecking the S+ (correct response) was rewarded by a 5-s access to the food hopper, while pecking the S− (incorrect response) caused the key to switch off with no hopper access. Following a pecking key presentation, the bird had to leave the perch, either to peck the key or not, and return to it to trigger the next trial. No inter-trial interval was imposed, neither after a correct response, nor after an incorrect response. Hence, the cost of incorrectly pecking the S− was that associated with a return-trip from perch to feeder, and operating the pecking key, without any opportunity to feed. Learning the S+/S− discrimination improved across trials, and all birds gradually reached our performance criterion (see below), so they were clearly motivated to learn without any additional punishment on incorrect responses. Performance was calculated automatically by the computer every 20 trials. When the bird reached 90% correct responding (pecking the S+ and withholding from pecking the S−) on two successive blocks of 20-trials, the predictive value of the S+ and S− was automatically reversed by the computer, such that the next pecking key presentation requested by the bird exposed it to the reversed contingency.

#### Reversal learning

Training on the reversed cue contingency continued until birds reached a 90% criterion on two successive 20-trial blocks, at which point, the computer controlling the operant conditioning equipment immediately reversed the predictive value of the colour cues once again. In total, each bird completed four successive cue reversals. At the end of testing the birds were returned to group housing.

### Analyses

To obtain a measure of innovation performance for each bird, we calculated the mean solving latency across the three innovation tasks for each bird. To examine the relationship between innovation propensity and learning ability, we correlated the mean innovation latency with the total number of blocks to reach criterion on the initial S+/S− discrimination using a Spearman rank correlation. As we were interested in examining the relationship between each bird’s ability to innovate and its ability to learn, and to reverse respectively, we calculated a reversal score that expressed each bird’s ability to reverse as a function of its ability to learn the initial S+/S− discrimination. For each bird and each reversal, the reversal score was the ratio between the number of blocks the individual had taken to complete the reversal and the number of blocks it had taken to complete the initial discrimination. In this way, for example, a bird that took twice as many blocks to reverse than it did to learn the initial discrimination was considered a faster reverser than a bird that took three times more blocks to reverse than it did to learn the initial discrimination. However, two birds with equal reversal speeds, but different learning speeds, were considered to have different reversal abilities. This reversal score has been used in the past to demonstrate between species differences in reversal performance [40], and applies the same logic as other attempts to examine the relationship between behavioural traits, and learning and reversal learning, respectively [21]. To examine the relationship between innovation propensity and behavioural flexibility, we conducted four planned non parametric Spearman rank correlations between each individual’s mean innovation latency and its reversal score for each of the four successive reversals. All statistical analyses were conducted on SPSS 20 (SPSS Inc., Chicago, IL, U.S.A.). All tests were conducted using two-tailed significance thresholds set at 0.05.

## Results

Mean (± SE) solving latency across the three innovation tests was 1138 s ±125 s. Solving latencies did not differ significantly across tests (mean ± SE: test 1∶1024 s ±199 s; test 2∶1261 s ±173 s; test 3∶1130 s ±205 s; paired samples Wilcoxon signed rank test, P = 0.584). Neither solving latency, nor solving success differed significantly across the five different extractive foraging tasks (latency: independent samples Kruskal-Wallis test, N1 = 10, N2 = 10, N3 = 8, N4 = 8, N5 = 18, P = 0.415; success: Fisher exact test, P = 0.674). Solving latencies were not correlated across tests (all N = 18; test 1 vs test 2, Spearman’s rho = −0.174, P = 0.489; test 2 vs test 3: Spearman’s rho = 0.314, P = 0.205; test 1 vs test 3: Spearman’s rho = 0.269, P = 0.280).

All birds increased the number of correct responses (pecking the S+ and withholding from pecking the S−) across trials both while learning the initial discrimination, and while learning each of the four reversals. All birds eventually reached criterion and progressed to the next stage (e.g. from the initial discrimination to the first reversal). The median number of 20-trial blocks to learn the initial S+/S− discrimination was 14. Subsequent reversals 1 to 4 took 19, 26, 25 and 25 20-trial blocks respectively. Reversal scores were highly significantly positively correlated across successive reversals (R1 vs R2, N = 18, Spearman’s rho = 0.761, P<0.001; R2 vs R3, N = 16, Spearman’s rho = 0.709, P<0.001; R3 vs R4, N = 15, Spearman’s rho = 0.696, P<0.001).

**Figure 2:**
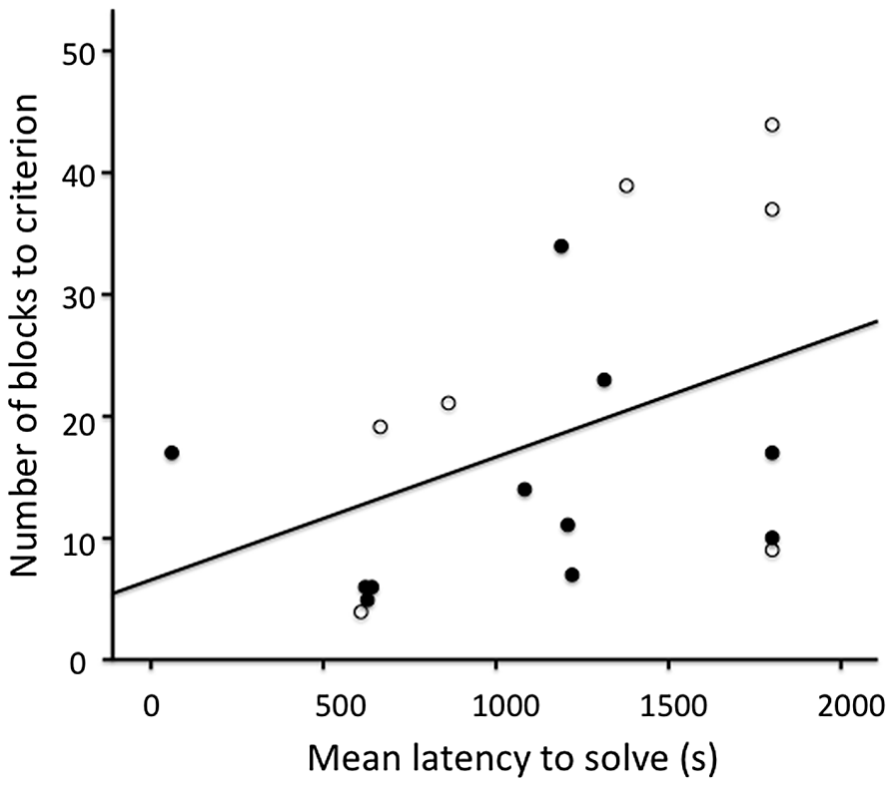
Relationship between innovation performance and discrimination learning. Innovation performance was calculated as the mean latency to solve three different extractive foraging tasks (see Figure 1). Learning performance was measured using the total number of 20-trial blocks to reach a learning criterion (see text for more details). Open circles indicate female mynas, filled circles indicate male mynas.

**Figure 3:**
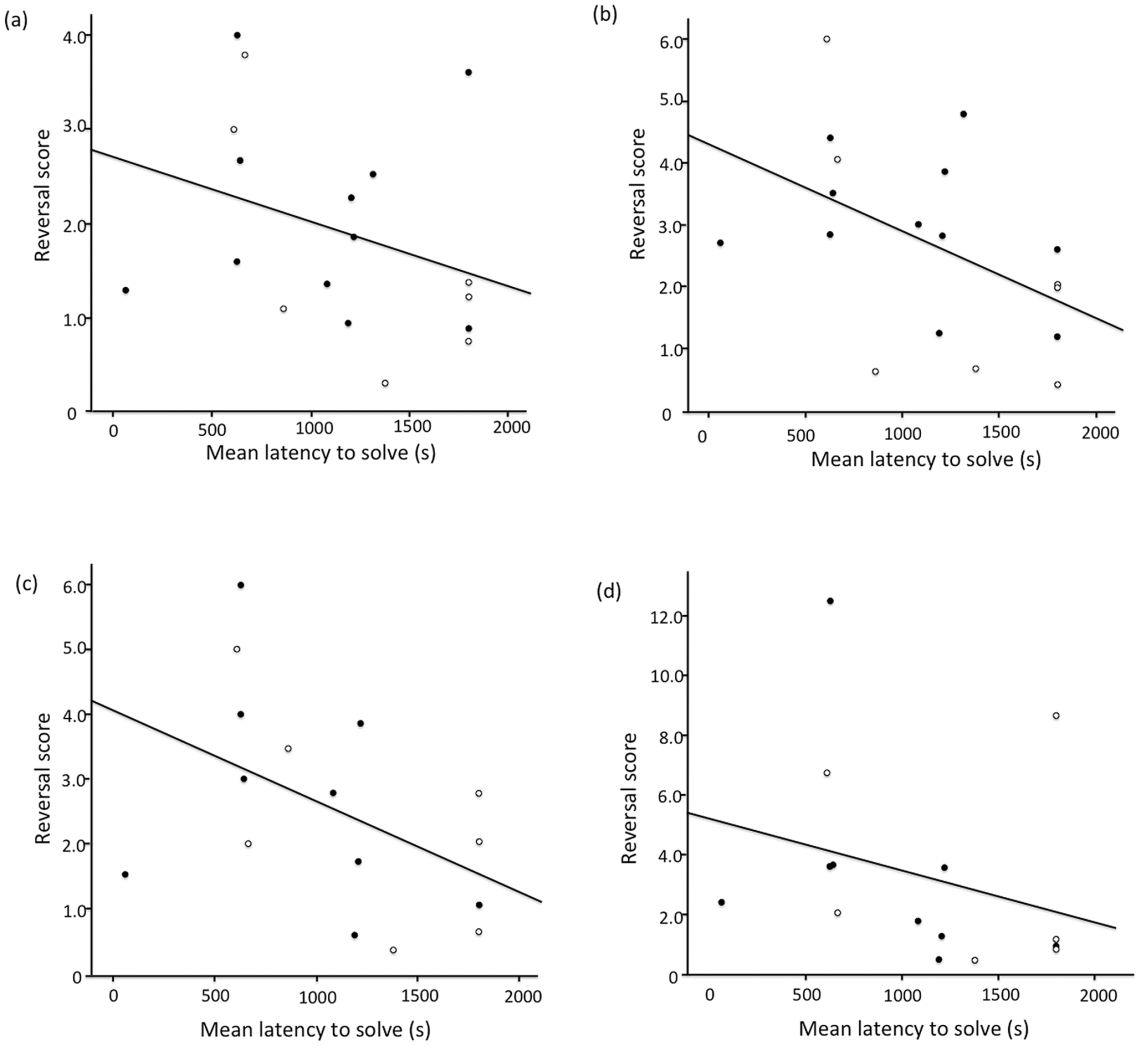
Relationship between innovation performance and reversal performance. Each panel depicts this relationship for one of four successive reversals. Innovation performance was calculated as in Figure 2. Reversal performance was measured using a reversal score, expressed as the total number of 20-trial blocks to reach criterion on a given reversal relative to the total number of 20-trial blocks to reach criterion on the initial discrimination (see text for more details). Open circles indicate female mynas, filled circles indicate male mynas.

Across birds, mean latency to innovate was significantly positively correlated with the total number of 20-trial blocks to learn to discriminate between the cue that predicted food (S+) and the cue that predicted no food (S−) (N = 18, Spearman’s rho = 0.499, P = 0.035; Figure 2), indicating that faster innovators learnt the initial discrimination between S+ and S− more quickly. In contrast, mean latency to innovate was significantly negatively correlated with the reversal score for three of four subsequent reversals (Spearman’s correlations: reversal 2, N = 18, coefficient = −0.547, P = 0.019; reversal 3, N = 16, coefficient = −0.516, P = 0.041; reversal 4, N = 15, coefficient = −0.523, P = 0.045; Figure 3). The correlation between each bird’s mean latency to innovate and its reversal score for the first reversal was in the same negative direction, but fell just short of significance (N = 18, Spearman’s rho = −0.410, P = 0.091). Total number of 20-trial blocks to learn to discriminate between the S+ and the S− was highly significantly negatively correlated with the reversal score for each of the four reversals (Spearman’s correlations, all P<0.001). These results supported our first prediction that faster innovators would learn a cue discrimination faster. Contrary to our second prediction, however, faster innovators were slower to change their behaviour in response to a changing environment.

## Discussion

A large body of comparative work has linked cross-taxon variation in innovativeness to an increased ability to adjust to novel and/or changing environments [4], [41]. Using an experimental approach, our research evaluated whether, similarly, inter-individual variation in innovativeness could be linked to an increased ability to adjust to a changing environment. Results revealed that although more innovative mynas learnt to discriminate between a signal for food and a non-signal for food more quickly, they were slower to change their behaviour when the significance of the food cues changed. This finding suggests a dissociation between the functional significance of inter-individual variation in innovativeness and variation occurring at higher taxonomic levels.

We found that faster innovators learnt more quickly to discriminate between a cue that signaled food and one that did not. This finding corroborates the conclusions from several earlier studies pointing to a link between innovation propensity and learning ability. Bouchard and Lefebvre [36] reported a positive relationship between innovation and social learning in pigeons (*Columbia livia*), while Overington et al [37] found that carib grackles (*Quiscalus lugubris*) with shorter innovation latencies were faster to learn to solve the problem across subsequent repeated presentations. Similarly, performance on an asocial learning task predicted innovation propensity in European starlings (*Sturnus vulgaris*) [35]. This consistently positive relationship between innovation and learning ability is behind the assumption amongst some authors that innovative behaviour is a measure of individual variation in cognitive ability [18], [26], [42], [43], as appears to be the case at higher taxonomic levels (3).

Although there is no universally accepted definition of intelligence, there is a convergent view that flexibility is one of its hallmarks [44]–[46]. Consequently, if innovation measures cognition, it should not only predict learning, it should also predict flexibility. Our finding that faster innovators were slower to reverse their behaviour when the environment changed is at odds with this conclusion and remains to be explained.

We speculate that individual innovation propensity may be associated with a collection of traits that belong to a broader pace-of-life syndrome. Indeed, their fast, but inflexible learning makes innovators akin to proactive individuals, while slow, but flexible learning aligns non innovators with a reactive personality [47]–[49]. As predicted by Sih & Giudice [49], fast, inflexible mynas may be favoring speed over accuracy relative to slow, but flexible individual mynas. Key to innovation may be perseverance, which would be advantageous in temporally and/or spatially stable, predictable environments, while more slow, but flexible behaviour may be advantaged in unstable, unpredictable environments, as has been proposed for other personality traits [21]. Spatial and temporal ecological variability may act to maintain individual variation in innovation propensity within a given species. Species with large variation along innovativeness and its associated personality traits would be able to adjust to a broader range of habitats, which would in turn yield marked population differences.

We found that learning was consistently related to reversal performance, and that reversal performance was stable across successive reversals. In other words, mynas were consistent in their learning and reversing performance. This finding supports the idea that fast-inflexible and slow-flexible learning are stable individual characteristics in mynas. In contrast, mynas were not consistent in the latency with which they solved across the three innovation tests, casting doubt on the suggestion that innovation propensity may be a stable individual characteristic, even though mean innovation performance was correlated with learning and flexibility. Yet in previous work specifically designed to assess inter-individual stability in innovation performance in mynas, we have found that innovation performance is repeatable across individual mynas [50], [51]. It is important to note our three innovation tests encompassed performance on five different innovation tasks with some individuals solving some tasks and other mynas solving others. High variability in innovation task, a relatively small sample size and the capped nature of the solving latency variable may explain why stability in innovation was not apparent in the present data set.

Where does a differential link between innovation and learning on the one hand, and innovation and flexibility on the other, leave the relationship between innovation and cognition at the inter-individual level? We suggest that both fast and flexible learning may depend upon neural processing power (e.g. neural volume, neuronal connectivity, neuronal density [44]). Innovation and reversal learning would hence capture two separate dimensions of cognitive ability, each with links to a different collection of personality traits. In this line of reasoning, the well-documented positive relationship between innovation rate, reversal learning and relative brain size at higher order taxonomic levels [4] would be underpinned by the existence of both fast, inflexible and slow, flexible phenotypes within a species with high neural processing power, and only slow, inflexible phenotypes within a species with low neural processing power. This idea would explain why a positive relationship between innovation and flexibility exists at the cross-taxon level, but appears to be absent at the inter-individual level.
